# Hydration Processes of Four-Component Binders Containing a Low Amount of Cement

**DOI:** 10.3390/ma15062192

**Published:** 2022-03-16

**Authors:** Iwona Wilińska, Barbara Pacewska, Valentin Antonovič

**Affiliations:** 1 Faculty of Civil Engineering, Mechanics and Petrochemistry, Institute of Chemistry, Warsaw University of Technology, Lukasiewicza Street 17, 09-400 Plock, Poland; barbara.pacewska@pw.edu.pl; 2 Laboratory of Composite Materials, Institute of Building Materials, Vilnius Gediminas Technical University, Linkmenų Street 28, 08217 Vilnius, Lithuania; valentin.antonovic@vilniustech.lt

**Keywords:** hydration, blended cement, pozzolan, fly ash, spent aluminosilicate catalyst

## Abstract

Results of research on hydration of four-component binders containing very high amounts of supplementary cementitious materials were presented. The samples were composed of blended pozzolana (a mix of conventional fly ash and spent aluminosilicate catalyst), cement (about 20 wt.% in the binder) and Ca(OH)_2_. Spent aluminosilicate catalyst was proposed as activating component which can improve properties of low-cement blends, while the role of Ca(OH)_2_ was to enhance pozzolanic reaction. Early and later hydration periods of such blends were investigated by calorimetry, TG/DTG, FTIR and X-ray diffraction. Initial setting time as well as compressive strength were also determined. It was concluded that enhancement of reactivity and improvement of properties of fly ash–cement binders are possible by replacing a part of fly ash with more active fine-grained pozzolana and introducing additional amounts of Ca(OH)_2_. The spent catalyst is mainly responsible for accelerating action during the first hours of hydration and for progress of early pozzolanic reaction. Fly ash develops its activity over time, thus synergic effect influences the later properties of composites. Samples containing blended pozzolana exhibit shorter initial setting times and higher compressive strength, as well as faster consumption of Ca(OH)_2_ compared to the reference. Investigated mixtures seem to be promising as “green” binders, alternatives to cement, after optimizing their compositions or additional activating procedure.

## 1. Introduction

Cement is a commonly used binder in cement concrete manufacturing, but CO_2_ emission is inherent in its production mainly due to a high-temperature process of cement clinker formation. Consumption of high amounts of energy and natural resources also takes place [1,2,3]. This is why various types of alternative cement clinkers and binder mixtures that are more ecologically friendly are in demand [2,4]. Reduction of usage of Portland cement clinker by its partial replacement in the binder/concrete composition with different kinds of mineral cement substitutes is the practice which has been applied for many years. Fly ash is a widely used supplementary cementitious material (SCM). Its typical content in a cement binder does not exceed 35 wt.% [5]. However, research work aimed at the application of significantly greater amounts of fly ash as cement replacement, even to 70–80% (very high-volume fly ash mixtures/concretes—very HVFA mixtures/concretes), has also been undertaken [6,7,8,9,10,11,12,13,14,15]. Using such high amounts of SCM as a cement substitute in the binder may solve, to some degree, problems connected with high demand for cement clinker and its environmental impact. However, replacement of high amounts of cement by fly ash, reactivity of which in the presence of water is significantly lower compared to cement, generates other disadvantages concerning properties of the final cementitious material. The main inconveniences are as follows: retardation of setting and hardening, thus lengthening of initial setting time, and slow growing of strength [5,16,17]. Despite the fact that fly ash develops its activity over time (in presence of cement and water), compressive strength of very HVFA composites may be reduced, often significantly, even after a long period of time [5,9,16,17,18].

Disadvantages described above can be explained by physical and chemical processes occurring in hardening binders containing very high amounts of SCMs. It is well known that fly ash undergoes chemical reactions in the presence of Ca(OH)_2_ and water (pozzolanic reaction). As a result, hydrated products, such as calcium silicates C-S-H, calcium aluminosilicates C-A-S-H, and calcium aluminates C-A-H, similar to those that arise during cement hydration, are formed [19,20] (cement chemistry notation: C—CaO, S—SiO_2_, A—Al_2_O_3_, H—H_2_O). In the case of low-calcium fly ash (Class F) used in the very HVFA mixture, the amount of Ca(OH)_2_ may be insufficient to develop fly ash pozzolanic activity to a high extent; many grains of fly ash are left unreacted in the paste and such a system is not able to achieve high compressive strength [15,17]. Further, in the case of high amounts of fly ash and low amount of cement, a fall of pH of pore solution decreases solubility of amorphous silica and alumina contained in fly ash grains [21,22]. Finally, the small content of cement cannot provide sufficient amounts of hydration products necessary to obtain the high strength of the ash–cement composite.

Therefore, research work of which the goal is to activate the system and optimize composition of mixture is carried out. As a result, an improvement of properties of the final composite can be expected. The authors of [8,10,12,23,24,25] proposed an application of different chemical activators for blended cements containing 70 or even 80% of Class F fly ash. In our previous paper, research results related to mechanisms of action of Na_2_SO_4_ and Ca(OH)_2_ used together to activate the fly ash–cement paste were described [13]. Different ways of activation of binding mixtures containing high and very high amounts of fly ash were discussed in the review paper [15]. 

Using blended pozzolans is one of the methods which can improve properties of discussed mixtures. Such blends are composed of at least two pozzolanic materials. Fly ash is one of them (it is the main component of the blend) and more active fine-grained pozzolanic material (e.g., silica fume [17,26,27], nano-silica [17,28,29,30,31], metakaolin [27,32]) is the other. In such a composition, very active pozzolana can react in the early hydration period, during which fly ash is almost inactive. Moreover, fine grains of the active pozzolana can accelerate hydration of cement (nucleation effect). In this way, the start of setting can occur faster, and early compressive strength may be higher compared to fly ash–cement mixtures without additional pozzolanic components [17,26,27,29,30,32]. Fly ash shows activity and contributes to the development of strength and tightness in later periods. In a multicomponent system, the synergic effect of action of all their components can be observed. Using pozzolanic material of low activity with another material having high pozzolanic activity can result in enhancing the benefits of their using [33]. Thus, such binders can show improved properties compared to binary blends. However, in the case of a very high amount of fly ash and a low content of cement, the discussed way of activation is only effective to some limited extent because the amount of hydraulic component (cement in this case) in the mixture is still low. This is why such compositions need additional activation if the final properties of the resulting material are unacceptable. For example, providing an extra substrate for pozzolanic reaction or introduction of additional alkaline components may be considered.

In our previous work [14], activation of a binder containing 80% of fly ash and only 20% of cement was proposed. This action consisted in partial replacement of fly ash by highly pozzolanic fine-grained aluminosilicate material—spent catalyst from catalytic cracking in a fluidized bed (FBCC). This by-product from the petrochemical industry, because of its physicochemical properties, is very attractive as a component of binding mixtures. The spent catalyst was investigated by us previously in the context of the possibility of its use as an active additive modifying the properties of materials made out of Portland cement [34,35,36,37,38] and calcium aluminate cement [34,39,40,41,42,43]. Studies on this by-product and its use as a component of various binding mixtures, including geopolymers, have also been carried out by other research teams [44,45,46,47,48,49,50,51,52,53,54,55].

According to our knowledge, in the work [14], the spent catalyst was proposed as an activator of very HVFA binders for the first time. The influence of similar blended pozzolan (i.e., fly ash + spent catalyst) on properties of final composite and formed products of hydration and pozzolanic reaction was also investigated by the authors of [33,56,57,58,59], however in those works the quantity of blended pozzolana in the cement binder was lower and amounted to between 30 and 50% of binder mass. In this paper, which is a continuation of [14], new compositions of very HVFA binders containing FBCC were proposed.

Properties of the final composite, important due to the possibility of its use, are closely related to the physical and chemical processes taking place in the system. The aim of this work was to investigate physicochemical changes occurring during hydration of a four-component binder composed of blended pozzolana (about 80 wt.%), Portland cement and an additional alkaline component (Ca(OH)_2_). The composition of fly ash–cement samples was determined on the basis of the following assumptions: (1) a very low amount of Portland cement was used, and industrial by-products were the main components of the mixtures; (2) fly ash was partially replaced by FBCC to accelerate cement hydration, initiate pozzolanic reaction faster, and as a result accelerate setting and hardening processes and development of compressive strength; (3) Ca(OH)_2_ was added to enhance pozzolanic reaction, provide the availability of Ca^2+^ to this reaction at the early stage (i.e., before the appearance of the proper amount of Ca(OH)_2_ from cement hydration) and allow the constituents of the pozzolana to react more fully; (4) the main composition of the samples was constant, i.e., the amounts of blended pozzolana, cement, Ca(OH)_2_ and water were the same in all samples, but composition of the blended pozzolana was different to show the role of FBCC in the mixtures. 

Several instrumental methods were used: calorimetry, thermal analysis, infrared spectroscopy, and X-ray diffraction. Initial setting time of the blends, as well as compressive strength of blended pozzolan–cement mortars, were also investigated.

## 2. Materials and Methods

The following materials were used: fly ash from a pulverized combustion of hard coal (PK), spent catalyst from catalytic cracking in a fluidized bed (FBCC), commercially available Portland cement CEM I 32.5 R (PC) produced by Cement Ożarów (Ożarów, Poland), as well as Ca(OH)_2_ of analytical grade. Average chemical compositions (given as oxides) and pH of water extracts were as follows:

- For PK: SiO_2_ 50%, Al_2_O_3_ 20%, Fe_2_O_3_ 7%, CaO 5%, SO_3_ 1%, Na_2_O + K_2_O 3.5%, MgO 3%, pH = 11.3 [14,60,61];
- For FBCC: SiO_2_ 50%, Al_2_O_3_ 40%, Fe_2_O_3_ < 2%, CaO 0.5%, SO_x_ < 3%, Na_2_O + K_2_O < 0.5%, MgO 0.5% [14,42], pH = 5.9;
- For PC: SiO_2_ 20%, Al_2_O_3_ 4%, Fe_2_O_3_ < 2.5%, CaO 63%, SO_3_ 3%, Na_2_O + K_2_O < 1.5%, MgO 2.5%, pH = 13.7.

Some other results for PK and FBCC (e.g., X-ray patterns, IR spectra) may be found in [43,60,61].

Fly ash–cement pastes similar to those that were studied in [14], i.e., containing about 20 wt.% of PC and about 80 wt.% of blended pozzolana (PK + FBCC), were investigated. In this work, an additional amount of solid Ca(OH)_2_ (10 wt.% in an amount of over 100% by weight of dry binder) was introduced. Water to solid ratio was 0.5 for each sample. Compositions of binders are presented in Table 1.

Dry components of binders (PK, FBCC, PC and Ca(OH)_2_) were mixed by hand to obtain a visually homogeneous form, then water was added, and samples were mixed again. The pastes were put in polyethylene forms, then they were closed and stored at room temperature. After an appointed period of hydration, the samples were removed, grinded and hydration processes were stopped using acetone. After the procedure they were investigated by TG/DTG, FTIR and XRD. Samples subjected to calorimetric measurements were hydrated in a calorimeter for 48 h. In the case of measuring initial setting time, mixtures were poured into a Vicat ring and the measure started from the moment of water adding.

Fly ash–cement mortars were made from binders of compositions presented in Table 1 and standard sand (450 g of binder, 1350 g of standard sand). Water to binder ratio was 0.5. The ingredients were mixed in a laboratory mixer and then the mixtures were poured into the molds (4 × 4 × 16 cm) and compacted in two layers by vibration. Then, the samples in the forms were covered with polyethylene foil to prevent water evaporation. The mortars were demolded after 2 days, then they were cured in water at room temperature.

Methods of investigations:

- Calorimetry—heat released starting from the moment of water adding until 48 h of hydration was measured by BMR calorimeter constructed at the Institute of Physical Chemistry, Polish Academy of Sciences (Warsaw, Poland). Samples were hydrated in a calorimeter at the temperature 25 °C. Dry mixture (10 g) and water (5 g) were first thermostated, then water was added to dry binder and the measurement started. The results were calculated using computer software [62].
- Thermal analysis—curves of TG and DTG were recorded using SDT 2960 Thermoanalyser (TA Instruments, New Castle, DE, USA) at temperature range 20–750 °C (heating rate 10 °C min^−1^) and at nitrogen atmosphere. The sample mass was 9–15 mg.
- FTIR spectroscopy—spectra were collected by FTIR spectrophotometer Genesis II (Mattson, Madison, WI, USA) at 4000–400 cm^−1^ wavenumbers (number of scans: 32, resolution: 4 cm^−1^). Samples were prepared as KBr pellets.
- X-ray diffraction—spectra were registered by HZG–4 diffractometer (VEB Freiberger Präzisionsmechanik, Freiberg, Germany), CuK_α_ radiation (anode voltage 30 kV, anode current 25 mA, 2θ range: 5 to 55°, step: 0.04°, time step: 4 s).
- Initial setting time was tested for pastes of compositions given in Table 1 using Vicat apparatus (MULTISERW, Marcyporęba, Poland), during the testing the samples were protected against water evaporation, the tests were performed basing on PN-EN 196-3:2016-12 standard.
- Compressive strength was measured for mortars after 28th and 90th day of hardening using strength machine ZD10/90 (WMW Fritz Heckert, Germany), the strength was measured according to PN-EN 196-1:2016-07 standard, six samples were tested for each composition, then the arithmetic mean was calculated.
- pH of water extracts 1:10 (one part of solid and 10 parts of water (by mass)) were measured by the use of CX 731 apparatus (ELMETRON, Zabrze, Poland). The pH values were registered in different times after addition of water to solid, starting at 5 min and ending at 60 min. Distilled water was used. The solution was being mixed all through the testing time.

## 3. Results and Discussion

### 3.1. Calorimetric Measurements and Initial Setting Time Testing

Several characteristic periods of heat release rate taking place one by one may be separated on a calorimetric curve of hydration of Portland cement and cement containing pozzolanic additives. This typical shape of calorimetric curve is well-known and was described in literature (e.g., in [14,18,19,20,38,63,64,65,66]). 

Replacement of a part of cement by fly ash results in the modification of heat release depending on the properties of fly ash and its amount in the mixture [15,67,68]. It is especially visible in the case of very high volumes of cement substitute. Exchange of high amounts of cement by conventional low-calcium fly ash leads to significant elongation of the induction period and lowering of the intensity of heat release compared to results for neat Portland cement [13,14,15,18,26,68]. Thus, in such pastes, delay of physicochemical processes of hydration takes place. It happens because: (1) the amount of hydraulic component is low, so-called dilution effect appears; (2) pozzolanic activity of fly ash is negligible at the early stages, (3) nucleation effect does not compensate the reduction of cement mass. The specific effect of fly ash depends not only on its amount and physicochemical properties but also on the conditions of hardening. Several factors, such as water to cement ratio, pH, and concentration of ions, also influence the early hydration of fly ash–cement mixtures [66]. For example, it was shown in [9] that an increase in water to binder ratio as well as an increase in fly ash replacement levels cause the elongation of initial setting time. The presence of FBCC also modifies the early hydration and properties of the mixture. In the case of blends containing FBCC, the availability of water for the hydration processes may be lower at the early stages because of high porosity of FBCC grains. However, this property means that the spent catalyst can be considered as a specific water reservoir in the tested system, thus it can be helpful in even distribution of water in the mixture. 

It has been shown in previous studies that the introduction of FBCC in an amount up to 25% into Portland cement paste causes an increase in exothermicity of the first wetting period (i.e., the period occurring just after addition of water) [34]. The amount of heat released by such a mixture in the initial time period is higher than in the case of reference cement paste, then, after a few hours, it becomes lower compared to the reference, which was also confirmed in studies described in [69] in the case of 35% cement substitution by the spent catalyst. Similar effects were also observed in the case of hydration of mixtures containing cement and natural zeolite [70,71]. Moreover, in both the cases, i.e., using FBCC or natural zeolite, the intensity of the second peak of heat release (corresponding to the hydration of alite) decreased with the raising amount of cement replacement level but the time of its occurrence also decreased (reduction of the induction period). It indicates acceleration of alite hydration in the presence of natural zeolite or FBCC as well.

**Figure 1:**
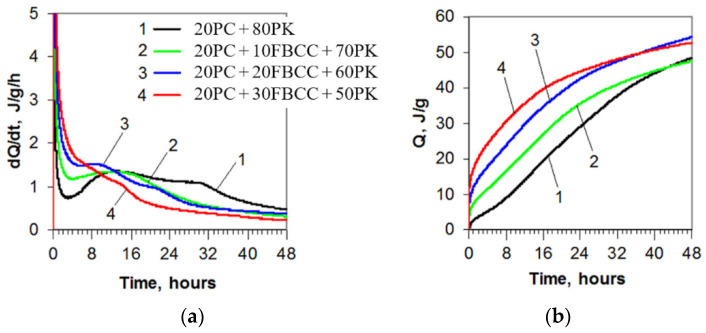
Heat evolution curves (**a**) and total heat released (**b**) for blended four-component binders.

In the case of cement pastes containing very high volumes of blended pozzolana composed of fly ash and FBCC, the influence of both the components on the early hydration process takes place. Calorimetric curves, presented in Figure 1, show that presence of FBCC in the investigated samples leads to acceleration of heat release, which was also observed previously [14]. As the content of FBCC increases, the effect relating to heat evolved in the wetting period becomes more intense and extended in time. Moreover, together with the rise of the FBCC amount, the induction period disappears. As a result of this, the beginning of the stage of crystallization of hydration products is difficult to observe (Figure 1a). In the case of the largest amount of FBCC in the sample (20PC + 30FBCC + 50PK), one extended in time effect of heat release is observed.

In general, the changes in kinetics of heat release in the case of the four-component pastes are similar to those that were registered for samples to which Ca(OH)_2_ was not added [14]. In the case of samples studied in this work, the processes of early hydration underwent without a clear separation of the induction period and this effect is more pronounced compared to results presented in [14]. It is the result of a complex impact of binder components on early hydration processes. Snellings et al. [72], who investigated zeolite + portlandite pastes, found that the presence or disappearing of the second peak of heat release depends on the kind of zeolite, including its fineness and the type of exchangeable cations, i.e., the properties which influence pozzolanic activity of zeolite [72,73].

**Figure 2:**
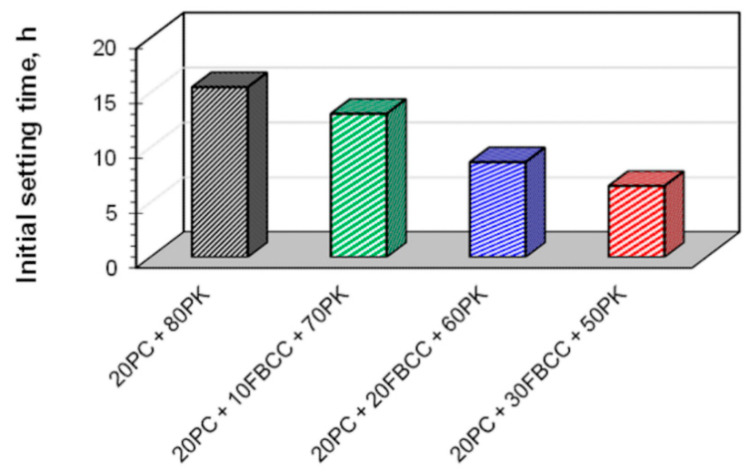
Initial setting time for blended four-component binders.

It is well known that replacing a part of cement by low-calcium fly ash may result in a delay of the initial setting time, and the effect is higher if more cement is substituted. The results of initial setting times (Figure 2) confirmed that exchange of 80% of cement in the mixture by fly ash results in elongated time of the start of setting despite the addition of Ca(OH)_2_. Using blended pozzolana is more favorable because of the significant reduction of initial setting time, especially in the case of high amounts of FBCC. The best results were registered for composition 20PC + 30FBCC + 50PK, for which initial setting time is more than two times shorter compared to the reference (20PC + 80PK). It confirms accelerating action of FBCC. On the other hand, it should be taken into account that the introduction of FBCC with its porous structure and highly developed specific surface area changes the workability of the mixture. 

**Figure 3:**
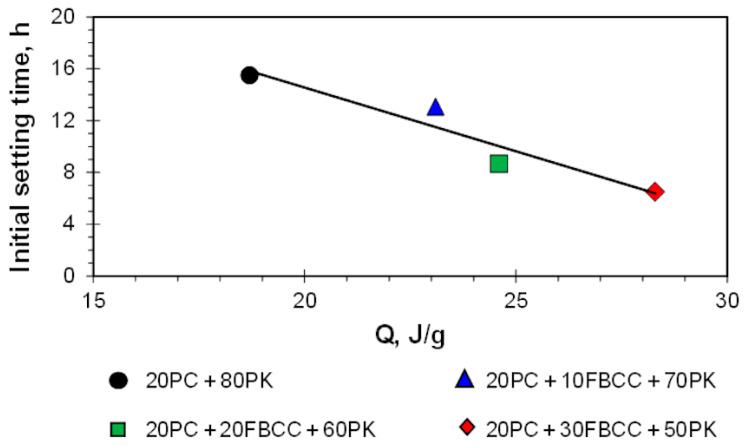
Dependence of initial setting time on heat released in the same period.

Figure 3 shows dependence of the initial setting time and total heat released registered to the moment of the start of setting for specific samples. It is evident that the greater the heat released, the shorter the initial setting time (the faster the start of hardening).

Considering the probable mechanism of early hydration of investigated binders, several possible processes which affect each other should be taken into account. Firstly, liquid phase quickly undergoes saturation with Ca^2+^ ions, generated by dissolution of Ca(OH)_2_ and by hydration of cement. pH of the solution rapidly reaches high alkaline values (pH of water extracts (one part of solid and 10 parts of water (by mass)) was 13.4 in the tenth minute after the addition of water into the solid mixture and 13.7 after 60 min in the case of sample 20PC + 80PK, similarly as for sample 20PC + 30FBCC + 50PK —13.6 for both after 10 and 60 min). Rapid heat generation in the first period of hydration is a consequence of the wetting of grains (especially porous grains of FBCC), as well as the dissolution of some components of the pastes (e.g., exothermic dissolution of Ca(OH)_2_). As cement hydration is exothermic too, it also contributes to generated heat. However, because the amounts of Ca(OH)_2_ and cement were the same for all samples, composition of blended pozzolana (i.e., the presence of FBCC in it) is mainly responsible for observed changes in the rate and intensity of heat release, as well as the rate of setting. Pozzolanic reaction is also exothermic [26,63,69], thus in the case of very active pozzolana it contributes to the amount of total heat released in the early hours of hydration.

As aforementioned, the more FBCC was added, the larger was the increase in early evolved heat being the result of wetting the grains of FBCC and sorption of water. During this process ions of Ca^2+^ can be adsorbed on active centers of FBCC grains [14,69], similarly as in the case of natural zeolite for which cation exchange in the first stage of pozzolanic reaction causes removal of Ca^2+^ ions from the solution [71,73,74,75] but the ions are not fixed [74]. It is also expected that, similar to the case of the natural zeolite, in the presence of FBCC some changes occur in unit-cell parameters and volume (the exothermal relaxation of the zeolite structure) as a result of rapid water adsorption [72]. With growing FBCC content, sorption of water by porous grains of FBCC causes a local reduction of the water/binder ratio. 

Processes which can accelerate or delay hydration take place almost at the same time. Cement hydration can be accelerated by an increase in the temperature of the sample (an effect of the increase in heat release in the presence of FBCC) and the nucleation action of fine grains of blended pozzolana. In this way, higher heat release is related to a faster start of hardening (shorter initial setting time). The disappearance of the induction period may indicate considerable acceleration of cement hydration, similar to the case of research described by authors of [76], who investigated hydration of alite in the presence of active silica. They observed an elimination of the dormant period as a result of the acceleration of hydration processes and rapid formation of the C-S-H phase [19,76]. On the other hand, the introduction of Ca(OH)_2_ may delay hydration of alite and belite, but FBCC grains can probably reduce Ca^2+^ ions from the solution. The obtained results indicate that in the case of the studied FBCC–fly ash–cement pastes, acceleration action prevails. 

After some hydration time, aluminate and silicate species, released from the aluminosilicate network of blended pozzolana and transferred into solution, undergo reaction with Ca^2+^. As a result, the formation of layers of the first products of pozzolanic reaction covering the grains of binder occurs [72]. Al-O bonds have lower bonding energy compared to Si-O and as a result they are weaker and more easily broken than Si-O [77]. Taking that into account one may expect that, in the presence of FBCC, aluminate ions transfer faster into a solution where they take part in reactions. As a result, formed initial gel is more enriched in aluminum. Small amounts of sulfate ions, introduced into the mixture mainly with cement, can be incorporated into C-S-H or C-A-S-H type products or they may form sulfoaluminates. 

Taking into account the capacity of FBCC grains for water sorption, one can expect that this water can be available at later periods of hydration processes. In such a way FBCC can act as a water reservoir and an internal curing agent, preventing autogenous shrinkage similar to natural zeolites [78]. Similar conclusions were drawn by the authors of the work [79].

### 3.2. Thermal Analysis

**Figure 4:**
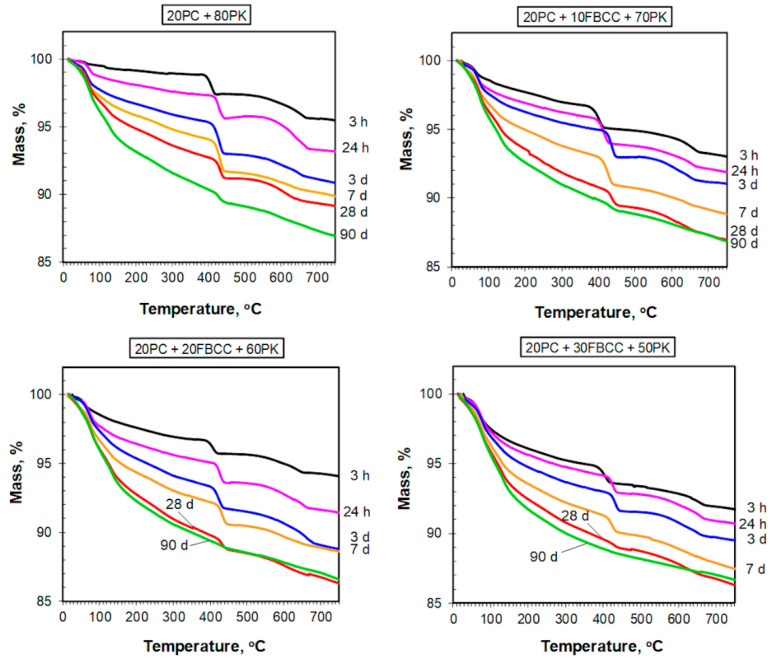
TG curves for blended four-component binders after different times of hydration.

**Figure 5:**
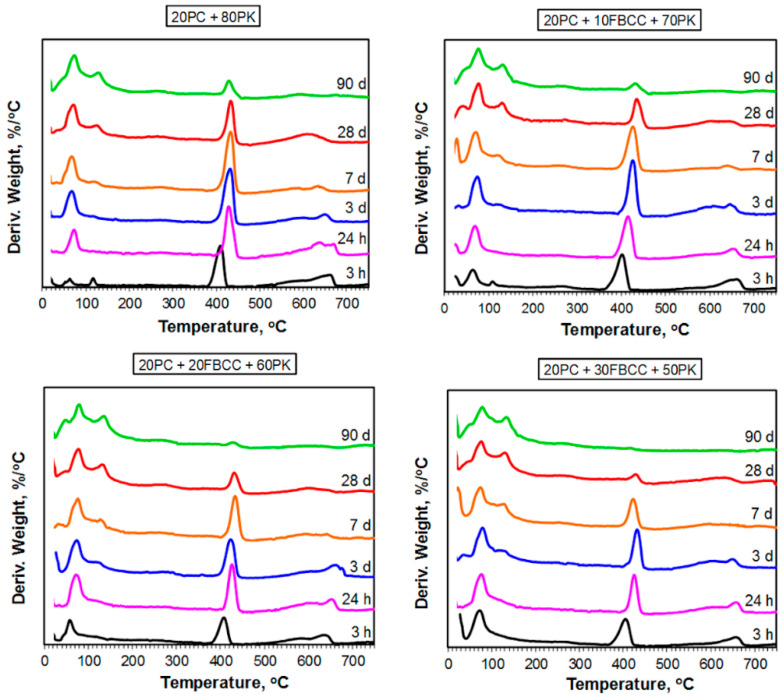
DTG curves for blended four-component binders after different times of hydration.

**Figure 6:**
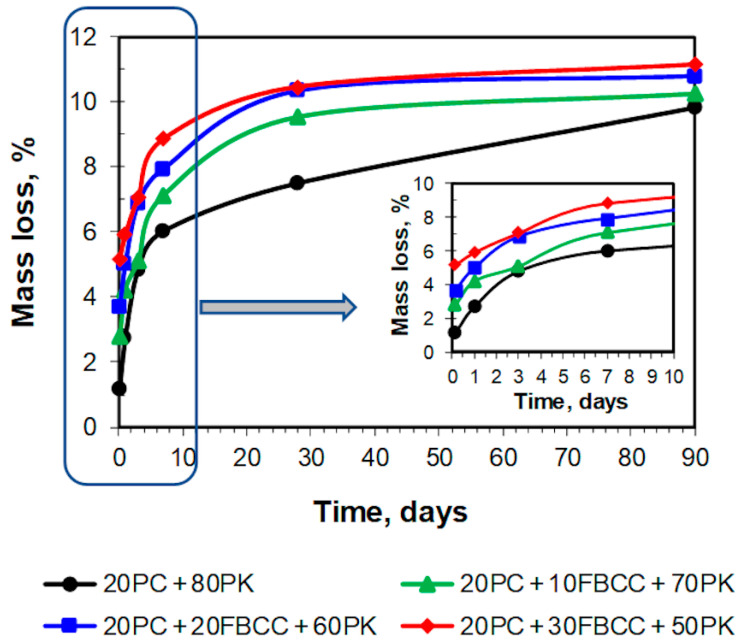
Mass loss relating to water bound in hydrates, such as C-S-H, C-A-S-H, sulfoaluminates, etc.

**Figure 7:**
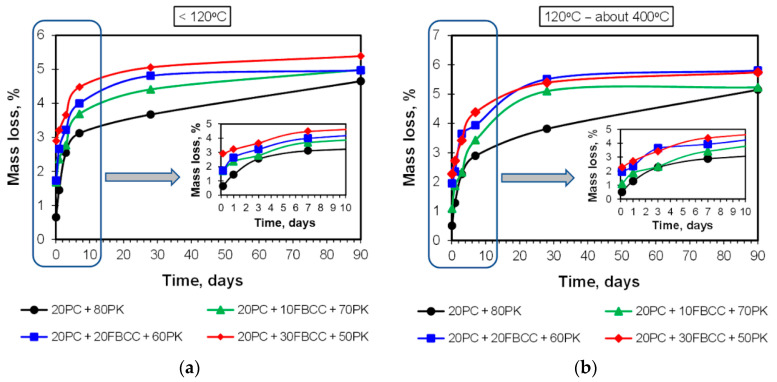
Mass loss at temperature range from 20 to 120 °C (**a**) and from 120 °C to the beginning of mass loss resulting from dehydroxylation of Ca(OH)_2_ (**b**).

**Figure 8:**
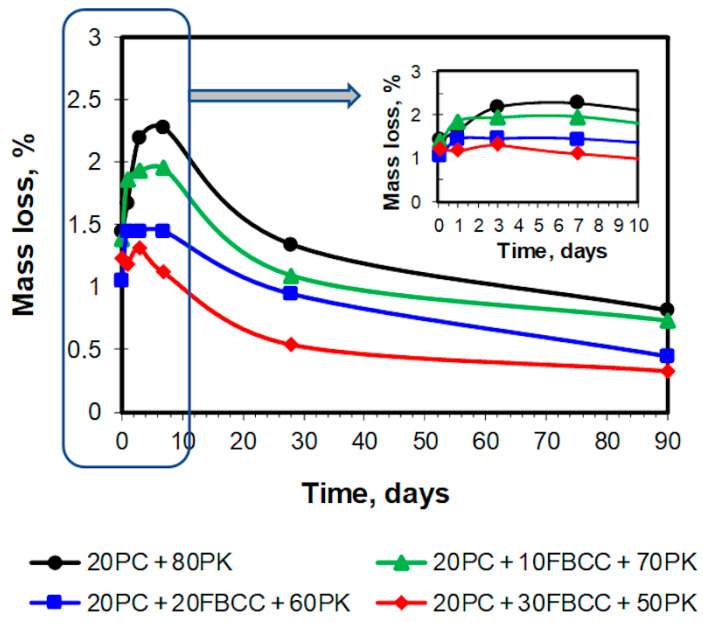
Mass loss corresponding to decomposition of Ca(OH)_2_.

**Figure 9:**
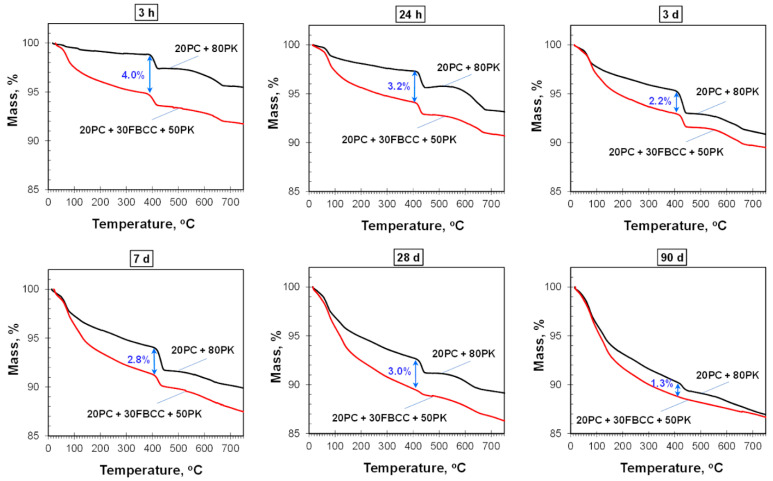
Comparison of TG curves obtained after different hydration periods for the reference sample (20PC + 80PK) and the paste containing the highest amount of FBCC (20PC + 30FBCC + 50PK), the differences between the amount of water bound in hydrates in both samples were indicated.

**Figure 10:**
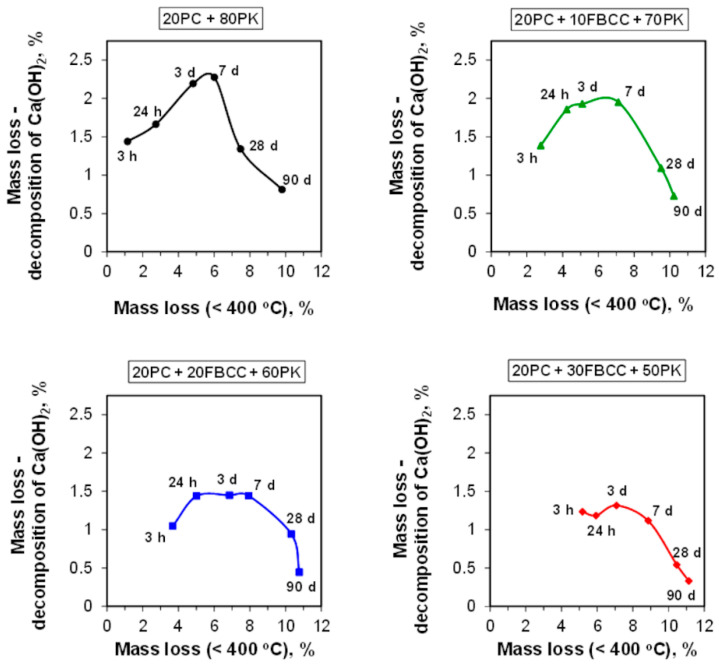
The dependence of the amount of water bound in hydrates and the mass loss resulting from the decomposition of Ca(OH)_2_.

TG and DTG curves registered after different hydration times are presented in Figure 4 and Figure 5 respectively. Diagrams of mass losses at specific temperature ranges are shown in Figure 6 and Figure 7 (the mass loss until about 400 °C—water bound in hydrates (bound water)), as well as in Figure 8 (the mass loss relating to decomposition of Ca(OH)_2_—the value proportional to the amount of Ca(OH)_2_ in the sample). Additionally in Figure 9, comparison of TG curves for the reference sample (20PC + 80PK) and the paste containing the highest amount of FBCC (20PC + 30FBCC + 50PK) is depicted to better show the differences in the development of bound water caused by the presence of FBCC. Moreover, Figure 10 presents the dependence of the amount of bound water and mass loss resulting from the decomposition of Ca(OH)_2_.

TG and DTG curves (Figure 4 and Figure 5) exhibit three typical mass losses, similar to the case of Portland cement paste:

- The first mass loss occurs until about 400 °C and covers dehydration of gypsum (at very early periods of hydration) and evaporation of bound water (water from products of cement hydration and pozzolanic reaction, i.e., hydrates type of C-S-H, C-A-H, C-A-S-H and sulfoaluminates);
- The second mass loss from about 400 °C to about 450 °C—decomposition of Ca(OH)_2_, which was introduced to the composition of samples (Table 1) and was also formed during cement hydration;
- The third mass loss above 500 °C—decomposition of carbonates (CaCO_3_ was introduced together with Ca(OH)_2_, fly ash and cement, and it is possible that in a small degree CaCO_3_ arose as a carbonation product during sample preparation).

In general, the courses of TG/DTG are similar for all the samples regardless of the composition of blended pozzolan. It indicates similar qualitative composition of investigated blends; however, quantitative composition is different. Changes observed over time of hydration indicate progress of hydration/pozzolanic processes.

#### 3.2.1. Results of TG/DTG at the Range of Dehydration of C-S-H, C-A-S-H and Sulfoaluminates

As it might be expected, the amount of water bound in hydrates (illustrated by TG course (Figure 4 and Figure 9) and mass loss at temperature range up to about 400 °C (Figure 6) is the smallest after 3 h of hydration, and it increases over time as a result of progress of cement hydration and pozzolanic reaction. The amount of bound water is higher for all samples containing FBCC in comparison to the reference mix throughout the studied period. Moreover, the greater amount of FBCC, the higher amount of bound water. A similar relationship was observed in the work [14]. The increase in bound water results from the porous structure of FBCC grains which absorb water, and also from acceleration of hydration processes, higher degree of reaction, and faster development of pozzolanic transformation.

It can be seen that the differences in bound water resulting from the presence of FBCC depend not only on the compositions of the mixtures but also on the time of hydration (Figure 6 and Figure 9). This means that the amount of bound water is closely dependent on the physical and chemical processes affected by the presence of FBCC. Detailed analysis of the mass losses shows that, despite the fact that over the entire study period all samples containing FBCC have a higher amount of bound water compared to the reference, these differences in bound water decrease by the third day of hydration, and after reaching the minimum, they increase again (Figure 9). Then, over time (after the 28th day of hydration), the differences in the amount of bound water in the reference sample and mixtures containing blended pozzolan become smaller. In later periods of hydration (on 90th day) the amounts of water bound are similar for all the samples. For example, after 24 h of hydration the amount of water bound by the reference is about 2.2 times lower as compared to the result for the sample 20PC + 30FBCC + 50PK. On seventh day of hydration, the amount of bound water is about 1.5 times lower for sample 20PC + 80PK, while on 90th day it is only 1.1 times lower compared to the result for 20PC + 30FBCC + 50PK. Such a tendency of changes confirms participation of FBCC in the pozzolanic reaction and acceleration of cement hydration mainly in the early days. Then, development of PK pozzolanic activity at subsequent hydration periods takes place. Summarizing, the observed changes in the amount of bound water indicate that, in the initial periods after adding water to the binders, cement hydration is accelerated by FBCC, and later the rate of hydration decreases, unlike in the reference sample, where a gradual development of hydration is observed. Between the third and seventh day of hydration, the development of the pozzolanic reaction is more intense in the presence of FBCC. Then, between the 28th and 90th day of hydration, the participation of FBCC in the pozzolanic reaction decreases, and the fly ash’s pozzolanic activity develops further.

It is well-known that the mass loss related to bound water is connected with various hydration products, of which kinds and amounts differ over time. DTG shape indicates the presence of some hydrated phases (Figure 5). For the reference sample (20PC + 80PK) as well as for the one containing the lowest amount of FBCC (20PC + 10FBCC + 70PK), in the first investigated period of hydration (i.e., after 3 h), two low intensity effects may be separated on DTG at temperature range until 150 °C. Similar results were registered previously [14] for samples which did not contain additional amount of Ca(OH)_2_. The peak with an extreme at temperatures below 100 °C is present for all samples and for all investigated periods of hydration. Its intensity and position change over time. This effect, especially after the first day of hydration, corresponds mainly to the presence of C-S-H in the hydrated mixtures. For pastes which are comprised of blended pozzolana, the second effect on DTG registered at 3 h of hydration (at temperature about 110–120 °C) is weaker and it is visible only for the sample containing 10% of FBCC (unlike in the case of results presented in [14], where it was observed for all samples after 3 h of hydration). For all investigated mixtures, this peak on DTG is not observed after 24 h of hydration. This is connected with the depletion of gypsum and formation of hydrated products. It seems that in the presence of FBCC and easily available Ca^2+^ ions, transformation of gypsum into sulfoaluminate phases is accelerated. Incorporation of SO_4_^2−^ ions into a new formed C-S-H phase is also possible. Elevated temperature and high pH of the reaction environment favor this process [80,81]. 

DTG curves registered after 3 days of hydration for samples containing 20 and 30% of FBCC show appearance of a new peak at about 130 °C, indicating formation of phases of hydrated aluminates. This effect is almost invisible in the case of the sample containing the lowest amount of FBCC and for the reference. For these mixtures this effect appears on subsequent days. This once again indicates the participation of FBCC in early formation of hydrated phases. The discussed peak tends to shift towards slightly higher temperatures as time passes and the FBCC content increases. Moreover, this effect is pronounced more for samples containing FBCC in comparison to the reference. It confirms that, in presence of FBCC, aluminate ions are released more rapidly and in larger amounts than in the case of the reference mix; consequently, they are able to undergo reactions and form solid products. 

It is difficult to confirm the presence of individual hydrated components based on TG/DTG only, because dehydration processes overlap. However, at the temperature range in which bound water is released, some hydrated phases undergo dehydration mainly at lower temperatures, while other phases at higher temperatures, depending on their thermal resistance. One can assume that the mass loss observed up to 120 °C is mainly related to water arising from dehydration of C-S-H and ettringite, while dehydration of C-A-H and C-A-S-H phases takes place mainly at a temperature range from 120 to about 400 °C [82]. Figure 7 shows that the presence of FBCC results in a growth of mass loss in both of the temperature ranges. In the case of samples containing FBCC, the increase in water bound both in C-S-H as well as C-A-S-H and C-A-H type products over time is observed mainly until the 28th day of hydration. On the contrary, in the case of reference mix without FBCC, the continuous significant increase in these mass losses is observed also in later days. It confirms once again that FBCC promotes formation of silicate and aluminosilicate products on earlier periods compared to the mixture without this component. In early hydration days, bound water is mainly the result of cement hydration which is accelerated by FBCC. Then, pozzolanic reaction products also contribute to an increase in the amount of bound water. FBCC undergoes pozzolanic reaction faster compared to fly ash, because FBCC shows higher pozzolanic activity.

#### 3.2.2. Content of Ca(OH)_2_ and Carbonates

The mass losses connected with the decomposition of Ca(OH)_2_ (Figure 8) after 3 h of hydration are similar for all samples. In subsequent investigated periods of early hydration this mass loss increases, which indicates that in the first days after water adding, hydration of cement predominates over pozzolanic reaction. However, it should be emphasized that a clear rise of content of Ca(OH)_2_ with time is primarily visible for the reference as well as for the sample containing the smallest amount of FBCC (20PC + 10FBCC + 70PK). In the case of greater content of FBCC (20 and 30%) the mass loss values registered after 24 h and 3 days are similar, which may be the effect of early pozzolanic reaction. On the other hand, the presence of 20 and 30% of FBCC could result in some delay of initial cement hydration, resulting in lower amount of Ca(OH)_2_ formed. However, continuous increase in water bound in hydrates (Figure 6) confirms reactivity of the system, both pozzolanic reactivity and cement hydration. It is clearly visible in Figure 10, which depicts the dependence of the amount of bound water and mass loss resulting from decomposition of Ca(OH)_2_. The results show that in the case of the reference sample, bound water and the amount of Ca(OH)_2_ increase up to the seventh day of hydration. In the case of samples 20PC + 10FBCC + 70PK and 20PC + 20FBCC + 60PK, the amount of Ca(OH)_2_ is lower compared to the reference and almost does not change between 24 h and the seventh day, while the amount of bound water increases. It can be seen that 10% of FBCC accelerates early hydration of cement, while in the case of a higher amount of FBCC, pozzolanic reaction takes place at this early period.

For most samples clear development of pozzolanic activity, manifested by reduction of Ca(OH)_2_ content, takes place after the seventh day of hydration, but for the paste 20PC + 30FBCC + 50PK it is visible earlier, i.e., between the third and the seventh day. After 90 days of hydration the blend containing the highest amount of FBCC no longer presents the content of Ca(OH)_2_ (TG and DTG curves for 90th day of hydration, Figure 4 and Figure 5). Thus, the results confirm that the mixture containing FBCC exhibits pozzolanic activity faster, which is developed until the disappearance of Ca(OH)_2_.

Over the time of hydration, reduction of the effect typical for decomposition of carbonates also occurs, which can be observed on TG/DTG curves (Figure 4 and Figure 5). It is caused by a slow reaction of carbonates, resulting in the formation of carboaluminates for which dehydration can be expected up to 300 °C. Thus, the presence of these compounds in the samples can also contribute to the overall mass loss discussed in the Section 3.2.1 of this paper.

### 3.3. Infrared Spectroscopy

**Figure 11:**
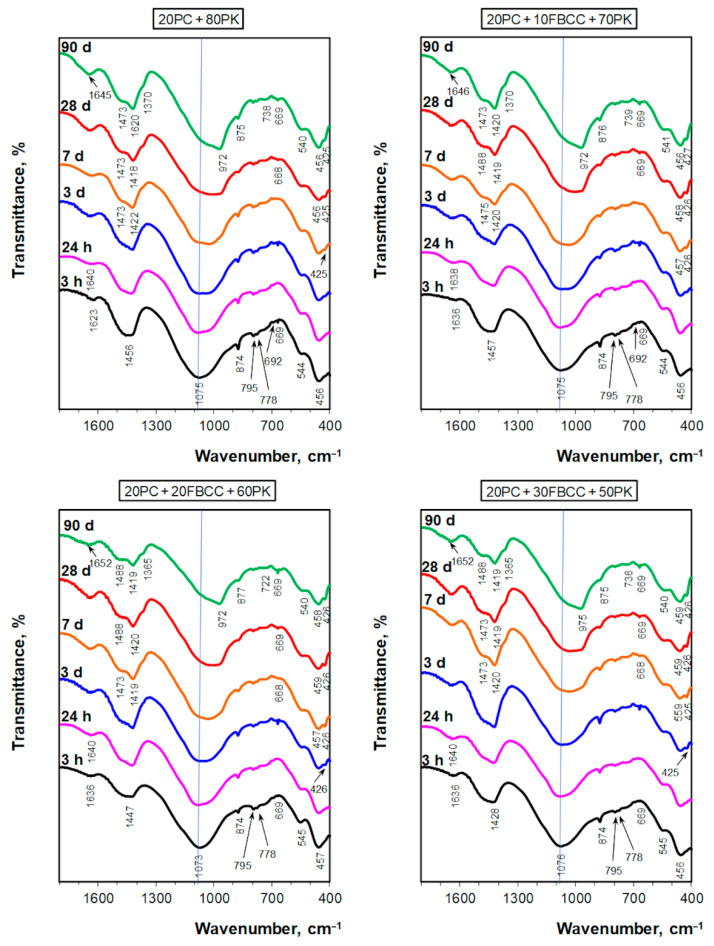
IR spectra for blended four-component binders after different times of hydration (at wavenumber scale from 400 to 1800 cm^−1^).

**Figure 12:**
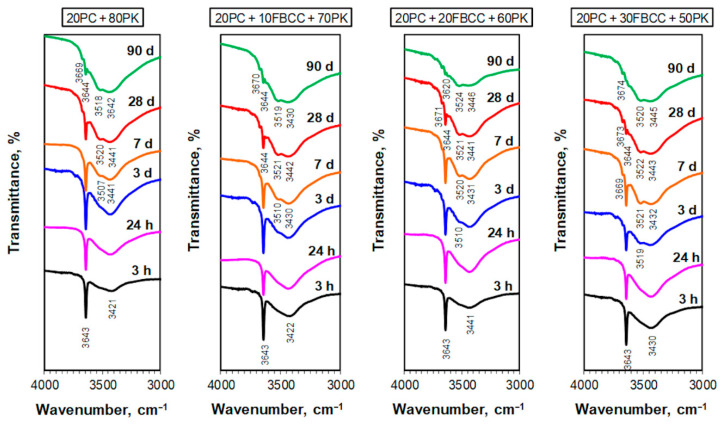
IR spectra for blended four-component binders after different times of hydration (at wavenumber scale from 3000 to 4000 cm^−1^).

IR spectra of investigated samples (Figure 11 and Figure 12), considered at the same period of hydration, are very similar regardless of the composition of the dry mixture of the binder. Thus, it confirms, similarly as TG/DTG results, that qualitative compositions of investigated blends are similar on a given day of hydration. 

The main intensive absorption band is situated at the range of wavenumbers between 900 and 1300 cm^−1^. Its extreme is at about 1075 cm^−1^ at early periods of hydration (Figure 11). This band corresponds to vibrations of Si(Al)-O bonds [77,83] resulting mainly from silica and aluminosilicate components in PK and FBCC. In this range of wavenumbers, several overlapping bands may be expected, i.e., the vibrational bands of quartz, mullite and vitreous phase [77,84]. There are several other smaller intensity bands resulting from the presence of PK in the investigated samples: doublet at about 795 and 778 cm^−1^ (quartz, symmetric stretching vibrations of Si-O-Si [85]); effect at 545 cm^−1^ (it may result from the presence of mullite [25,77,84]); and band at about 456 cm^−1^ (bending vibrations of O-Si-O (silicate tetrahedra) [85]). Weak, sharp band at about 670 cm^−1^, hardly visible band at 692 cm^−1^ and the clear band at about 456 cm^−1^, are probably also due to the presence of quartz in PK [84]. However, the band at about 670 cm^−1^ may be also connected with the presence of a small amount of gypsum [86,87,88]. Unfortunately, the position and intensity of the most intense band for blended pozzolana make it so that the main effects corresponding to sulfates (vibrations of SO_4_^2−^ at 1100–1200 cm^−1^ [87,89]) overlap with this band. For a similar reason, formation of the first portions of the C-S-H phase, which was confirmed in TG/DTG results, is not clearly visible in IR spectra after early hydration periods, because the typical position of the vibration band of silicate species in C-S-H is 970–975 cm^−1^ [90].

The band at about 1630 (Figure 11) and a broad one at 3100–3600 cm^−1^ (Figure 12) are connected with vibrations of water molecules—deformation and stretching vibrations, respectively. One intense sharp band at 3643 cm^−1^ corresponds to vibrations of OH in Ca(OH)_2_. 

Several absorption bands confirm the presence of carbonates: one at 1350–1600 cm^−1^ (the main carbonate band of medium intensity) and one at about 875 cm^−1^. 

Changes in IR spectra occurring over time confirm that hydration and pozzolanic reactions take place. 

Some deepening of the band of stretching vibrations of H-O-H molecules (at 3000–3600 cm^−1^) over time indicates progress in water binding into hydrated forms (Figure 12) and confirms conclusions from TG/DTG results. For mixtures with 20 and 30% of FBCC, on the third day of hydration, a few small additional effects at the region of 3100–3600 cm^−1^ may be separated, indicating the formation of new forms of hydrates. On the seventh day, as well as in subsequent days, there are additional bands visible in a wide band of stretching vibrations of H-O-H. This demonstrates the creation of new forms of hydrates. Bands at higher wavenumbers may be associated with stretching vibrations of SiO-H [91], while the one at about 3670 cm^−1^ may indicate formation of calcium monocarboaluminate [84,92] or hexagonal hydrates, e.g., C_4_AH_13_ [92].

Progress in physicochemical processes of hydration is also visible in the location of the band of deformational vibrations of water molecules. This band, starting from early periods of hydration, gradually shifts towards higher wavenumbers. Its maximum occurs at about 1625 cm^−1^ in the case of reference paste (after 3 h of reaction), while for samples containing FBCC at about 1635 cm^−1^ (at the same hydration period). It may indicate more advanced hydration in the presence of the spent aluminosilicate. After 7 days, the band’s location is at 1635–1645 cm^−1^, while after 90 days it is at 1645–1655 cm^−1^ (higher values are observed for samples containing 20 and 30% of FBCC). Such changes in the location of the band and its final maximum at about 1650 cm^−1^ are related to bending vibration of irregularly bound water [89] resulting from the progress of the hydration.

Some changes in the shape of the main band of pozzolana, i.e., the band of Si(Al)-O vibrations, and its shift are also visible. Just after the first day of hydration, a small widening of this band towards lower wavenumbers is observed, which proves the formation of new silicate or aluminosilicate forms. In the case of samples containing 20 and 30% of FBCC, a small new band at about 425 cm^−1^ appears on the third day. This band is related to vibrations of Al-O [88,92] and indicates the formation of hydrated aluminate or aluminosilicate phases. The appearance of this effect in the spectra has good correlation with the DTG results, which show the presence of hydrated aluminate phases on the third day of hydration for the samples 20PC + 20FBCC + 60PK and 20PC + 30FBCC + 50PK. 

IR spectra collected from the seventh day of hydration show a gradual appearance of reaction products, such as hydrated silicates and aluminates, more clearly. This is evidenced primarily by the changes observed in the shape of the main absorption band—it is shifting its extreme towards longer wavelengths (Figure 11). On the seventh day of hydration, the maximum of this band is at about 1030 cm^−1^. On this day, a band at about 425 cm^−1^ is visible for all samples—very weak for the reference and more noticeable for the blends containing FBCC. A band at about 670 cm^−1^ is also visible (for all samples after the 28th day of hydration). This band may be connected with bending vibrations of Si-O-Si in C-S-H gel [90,91,93,94], which is confirmed by its better visibility on later days of hydration. After 28 days, an intense band with a sharp maximum at about 970 cm^−1^ is observed for all samples. It confirms the formation of the C-S-H phase as a result of hydration of cement and pozzolanic reaction between Ca(OH)_2_ and reactive components of blended pozzolan. Such location of the main band of Si-O stretching vibrations is typical for C-S-H gel arising during cement hydration or synthesized in conditions of pH equal to 11 or higher [94].

The progress of the pozzolanic reaction with time is clearly demonstrated by a gradual reduction of intensity of the vibrational band of OH in Ca(OH)_2_ (sharp, thin band at 3643 cm^−1^, Figure 12). It is especially visible after the seventh day of hydration, which confirms conclusions from the TG/DTG results. More rapid disappearance of this band for samples containing FBCC indicates high pozzolanic activity of this additive. In the case of paste containing 30% of FBCC, after 28 days of hydration only very weak band at 3644 cm^−1^ confirms the presence of small amounts of Ca(OH)_2_. On 90th day, for a binder with the greatest amount of FBCC, presence of Ca(OH)_2_ is not visible. 

Changes in the shape of a band typical for carbonates are observed mainly from the seventh day of hydration. Separation of maximum at about 1420 cm^−1^ and shoulder at about 1475 cm^−1^ evidence transformations occurring in carbonate phases. Moreover, on the seventh day, a very small shoulder at about 1365 cm^−1^ begins to appear. A carbonate band in the range of 1330–1580 cm^−1^ loses its intensity, especially in the later stages of hydration (after 28 days). According to information provided by authors [89], such reduction in the intensity of the carbonate band may be partially caused by a reaction between calcium carbonate and aluminates with the formation of phases of carboxyaluminates. Moreover, CO_3_^2−^ ions may substitute sulfate ions in the Aft and Afm phases.

### 3.4. X-ray Diffraction

**Figure 13:**
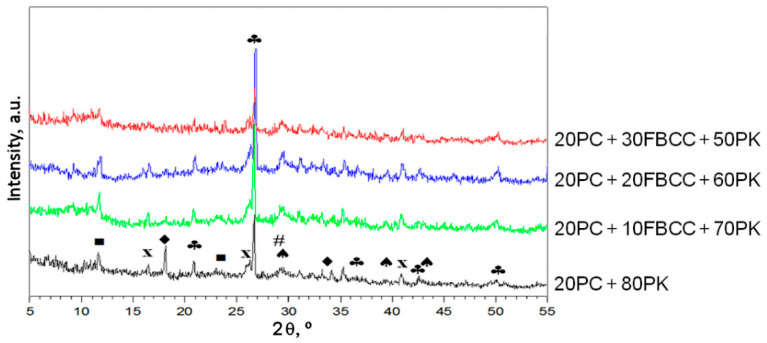
X-ray patterns for blended binder pastes (90th day of hydration); ♦ Ca(OH)_2_, ♣ SiO_2_, ♠ CaCO_3_, ■ monocarboaluminate, **x** mullite, **#** C-S-H.

X-ray diffraction patterns (Figure 13) confirm once again similar qualitative composition of all investigated samples. The results show that components of investigated pastes have a low degree of crystallinity despite long periods of hardening. Thus, products of hydration and pozzolanic reaction are rather difficult to identify in this way. The presence of Ca(OH)_2_ is visible only for the reference sample. It confirms the main conclusions from TG/DTG and IR results. For all samples there are a few bands resulting from the presence of quartz and mullite that confirm that a part of fly ash remains unreacted. Moreover, there are bands connected with carbonate phases: calcium carbonate and monocarboaluminate.

### 3.5. Compressive Strength

**Figure 14:**
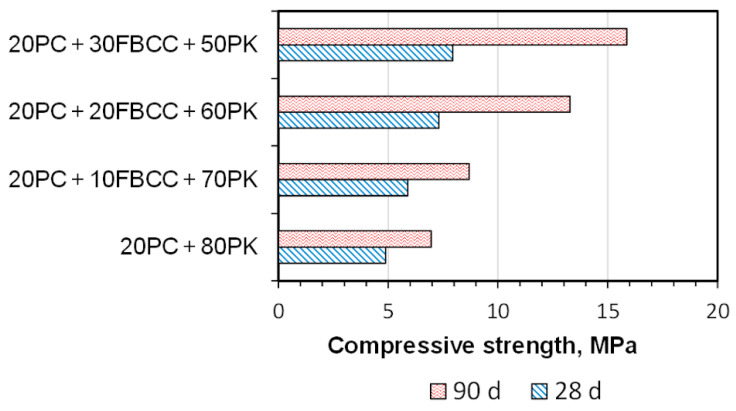
Compressive strength of blended binder mortars after 28 and 90 days of hydration.

Figure 14 presents compressive strength results for SCMs–cement mortars after 28 and 90 days of hydration.

Similar values of compressive strength of composites made with binders containing 70–80% of fly ash and water to binder ratio 0.4–0.5 were also obtained by the authors of [9,16,29]. It can be seen that compressive strength is the lowest for the reference sample. Over the time of curing, compressive strength of the sample 20PC + 80PK increases, which confirms development of hydration and pozzolanic processes. After 90 days the gain of compressive strength of this mix amounts to about 43% relative to the 28th day compressive strength. 

The presence of FBCC in the place of a part of PK results in a higher compressive strength of obtained mortars compared to the reference, both after the 28th and 90th day of hydration. After 90 days, compressive strength for mixes containing FBCC can be even more than two times higher (for sample 20PC + 30FBCC + 50PK) compared to reference at the same hardening period. Thus, it can be concluded that the replacement of PK by highly pozzolanic aluminosilicate material (FBCC) results in a faster development of compressive strength. It is visible on the 28th day of hydration, but the best results were registered at later hydration periods (synergic effect of FBCC and PK). Thus, one may conclude that partial replacement of fly ash by a spent aluminosilicate catalyst and the introduction of Ca(OH)_2_ is favorable to enhance properties of very high volume fly ash binder. To obtain higher compressive strengths of the mortars, the mixture composition and curing procedure should be optimized. For example, water demand and plasticizing admixture (if needed) should be determined experimentally, taking into account not only consistency of the fresh mixture but also the amount of water necessary for the pozzolanic reaction and hydration of cement. Using higher class Portland cement instead of 32.5 R cement and an elevated curing temperature can also be considered. However, to obtain a satisfactory level of compressive strength, additional activation techniques improving reactivity of the pozzolana may be needed, such as chemical activation with Na_2_SO_4_. These will be the directions for further research of such very HVFA systems.

## 4. Conclusions

The obtained results showed that there is a possibility to improve the properties of fly ash–cement binders performed with a small (about 20 wt.%) amount of Portland cement. The modification method consists of the replacement of a part of fly ash by more active fine-grained pozzolana (i.e., spent aluminosilicate catalyst) and the introduction of Ca(OH)_2_. 

The presence of an additional amount of Ca(OH)_2_ in the composition of the binder containing very high amounts of blended pozzolan results in a more complete pozzolanic reaction compared to the case where cement is the only source of Ca^2+^ ions. 

Kinetics of heat release for the binders containing a very high amount of blended pozzolana is different compared to typical result for Portland cement. An increase in the percentage of the aluminosilicate catalyst content results in emissions of more heat in the first periods of hydration and in the disappearance of the induction period. Hydration of cement is accelerated. 

In the presence of FBCC, Ca(OH)_2_ is consumed faster in a pozzolanic reaction. Moreover, more water is bound in hydrates, especially during early hydration.

The spent catalyst is mainly responsible for accelerating action during the first hours of hydration and for progress of the early pozzolanic reaction. Over time, fly ash reveals its pozzolanic activity and the final properties of the material are a result of the effect of both the components. 

Carbonates in investigated binders undergo a slow reaction with the formation of calcium carboaluminates in the later stages of hydration.

Qualitative compositions of investigated pastes after the same hydration stage are similar; however, quantitative compositions can be different. The differences are the most visible in early hydration days, then, over time, they disappear.

As a result of acceleration and the synergic effect of components of the blended pozzolana, initial setting time is shorter and compressive strength of composites is enhanced compared to results for reference specimens (without the spent catalyst). 

Investigated mixtures seem to be promising as “green” binder alternatives to cement. More favorable properties of the materials can be obtained after optimizing the blends’ compositions. Additional activation, e.g., by the introduction of an admixture of Na_2_SO_4_, can also be considered. These should be the directions for further research on these materials.

## Figures and Tables

**Table 1:** Compositions of investigated samples.

Samples	Content of the Components/g				
	Portland Cement (PC)	Fly Ash (PK)	Spent Catalyst (FBCC)	Ca(OH)_2_	Water
20PC + 80PK (reference)	20	80	0	10	55
20PC + 10FBCC + 70PK	20	70	10	10	55
20PC + 20FBCC + 60PK	20	60	20	10	55
20PC + 30FBCC + 50PK	20	50	30	10	55

## Data Availability

Data are contained within the article.
